# Thermal Conductivity of Polyisoprene and Polybutadiene from Molecular Dynamics Simulations and Transient Measurements

**DOI:** 10.3390/polym12051081

**Published:** 2020-05-09

**Authors:** Aleksandr Vasilev, Tommy Lorenz, Cornelia Breitkopf

**Affiliations:** Chair of Technical Thermodynamics, Technische Universität Dresden, 01069 Dresden, Germany; tommy.lorenz@tu-dresden.de (T.L.); cornelia.breitkopf@tu-dresden.de (C.B.)

**Keywords:** molecular dynamics simulations, force field, polyisoprene, polybutadiene, thermal conductivity, transient hot wire method

## Abstract

The thermal conductivities of untreated polyisoprene and polybutadiene were calculated by molecular dynamics (MD) simulations using a Green-Kubo approach between −10 °C and 50 °C at atmospheric pressure. For comparison, the thermal conductivities of untreated polyisoprene with a molecular weight of 54,000 g/mol and untreated polybutadiene with a molecular weight of 45,000 g/mol were measured by the transient hot wire method in similar conditions. The simulation results of both polymers are in good agreement with the experimental data. We observed that the MD simulations slightly overestimate the thermal conductivity due to the chosen force field description. Details are discussed in the paper.

## 1. Introduction

Polymers are excellent materials for developing new types of composites. This is due to their good mechanical properties and low mass. Polyisoprene and polybutadiene are widely used in rubber industry. These polymers have been chosen as building blocks for interactive-fiber rubber composites. Such new type of composites consist of a polymer matrix, reinforced fibers, shape-memory alloys and other fillers. To predict their thermo-mechanical behavior experiments as well as theoretical approaches are meaningful. For modeling of composites in a macro-scale, finite element methods (FEM) are widely used. To solve numerically conservation equations by FEM for them, one has to know the properties of each of their components. These properties can be obtained both from experiment like the transient hot wire method and from theoretical approaches, as for instance, MD simulations.

Much work has been done to describe the thermal conductivity of polymers by MD simulations [1,2,3,4,5,6,7,8,9]. It has been found a linear relationship between the thermal conductivity and the number of degrees of freedom per chain [7]. Thermal conductivities of polystyrene, stretched polystyrene, mixtures of polystyrene and carbon dioxide as functions of temperature and pressure have been calculated by reverse non-equilibrium molecular dynamics (RNEMD) [8].

The thermal conductivity can be calculated, e.g., by non-equilibrium MD simulations (NEMD) and equilibrium MD simulations (EMD). The NEMD simulations are based on Fourier’s law. To obtain the thermal conductivity by this method, the heat flux and the temperature gradient must be known. In the EMD approach, which is based on the application of the Green-Kubo formula, the thermal conductivity is obtained by integrating the heat flux autocorrelation function. The contribution of many-body interactions to heat flux and thermal conductivity is discussed in ref. [10]. The authors propose a new method for the calculation of atomic stress tensor, which is used to calculate heat flux. To test this, calculations of the thermal conductivities of butane, octane and polystyrene were performed using this definition of the heat flux and the definition used in the LAMMPS [11] package. It was found, that the thermal conductivity calculated by LAMMPS is slightly overestimated [11]. In addition, it was noticed that the contribution of dihedral interactions to the thermal conductivity of butane, octane and polystyrene calculated with LAMMPS [11] is much lower compared to the method using the new definition of heat flux.

Using MD simulations [2,3], it has been found, that the thermal conductivity of polymers increases with increasing molecular weight, which has also been observed experimentally [12,13]. In ref. [3] the dependence of the thermal conductivity of amorphous polyethylene on the degree of polymerization at normal conditions has been found by MD simulations. It has been observed, that if the degree of polymerization is less than 7, the process of heat transfer is dominated by a collision mechanism. Moreover, if chains consist of more than 140 monomer units, the process of heat transfer is governed by phonon transport.

However, there are only a few MD simulations that deal with the thermal conductivity of polyisoprene. In ref. [4], it has been calculated via the EMD approach. Interactions between atoms have been described by an adaptive inter-molecular reactive empirical bond order (AIREBO) potential. But the thermal conductivity has been overestimated by more than two times. In ref. [6] an influence of the number of cross-links between the polymer chains by sulfur bridges to the thermal conductivity of cis-1,4-polyisoprene has been found. Derived from MD simulations, the dependence of thermal conductivity from unidirectional strain agrees well with the experimental data. For soft and hard rubber, the influence of temperature on thermal conductivity at atmospheric pressure has been determined. Agreement with experimental data is rather qualitative than quantitative. Consequently, there is a great need to develop more accurate force fields for MD simulations and to test existing force fields for polymers and their derived blends.

The transient hot wire method is widely used for measurements of thermal conductivities of liquids [14,15,16]. The approach has been implemented for the determination of thermal conductivities of medium and high vinyl content polybutadiene with molecular weights 2600 g/mol and 100,000 g/mol in untreated and cross-linked states [17]. The method also has been used for the determination of the thermal conductivity of untreated and cross-linked cis-1,4-polyisoprene at high temperature and pressure ranges [18].

In [19] it has been shown by experiments, that thermal conductivities of pure cis-polyisoprene and pure trans-polyisoprene blends are almost equal, but they had differences in crosslink density and crystallinity. The pure trans-polyisoprene blend had the highest crosslink density and crystallinity, whereas the pure cis-polyisoprene blend was amorphous with the lowest crosslink density in comparison with all investigated blends. For mixtures of the pure cis and trans polyisoprene blends, the crystallinity and the crosslink denstity have been increasing with an increase of content of the trans-polyisoprene blend. Thermal conductivities of the mixtures have been found even higher than the thermal conductivity of the pure trans-polyisoprene blend, which had the highest crosslink density among all blends. To explain this phenomenon, the authors have cited ref. [20], that if the average distance between crosslinks is less than the range of elastic disorder (10 Å), the thermal conductivity increases with an increase of the crosslink density, and it decreases, if the average distance between crosslinks is less than the range of elastic disorder [19].

The aim of this work was to derive a data set of the thermal conductivity of untreated polyisoprene and untreated polybutadiene between −10 °C and 50 °C at atmospheric pressure from MD simulations and compare them to data from the transient hot wire method. The results are important to find accurate force fields for the calculation of the thermal conductivities of rubbers, which will be based on these polymers.

## 2. Simulation and Experimental Details

**Figure 1:**
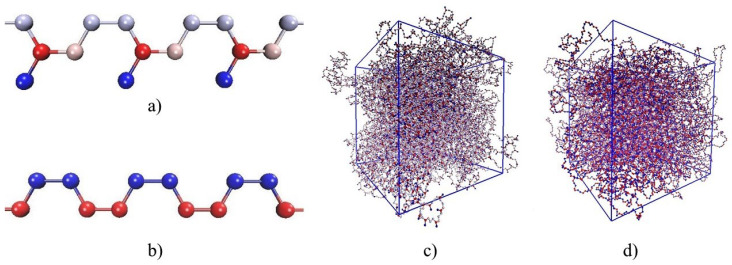
Models of polymeric systems: (**a**) fragment of cis-1,4-polyisoprene chain: C atom (red), CH group (pink), ${\mathrm{CH}}_{2}$ group (grey), ${\mathrm{CH}}_{3}$ group (blue); (**b**) fragment of cis-1,4-polybutadiene chain: CH group (blue), ${\mathrm{CH}}_{2}$ group (red); (**c**) 27 chains of polyisoprene in periodic supercell; (**d**) 27 chains of polybutadiene in periodic supercell.

To create input structures of the polymer systems the Moltemplate software [21] has been used. Firstly, two chains of cis-1,4-polyisoprene and cis-1,4-polybutadiene (see Figure 1) have been constructed, which consist of 200 monomer units. CH, ${\mathrm{CH}}_{2}$ and ${\mathrm{CH}}_{3}$ groups have been each modeled as one atom. A force field approach was needed to describe the interactions between the atoms. According to this, the total potential energy of a polymeric system is calculated to

(1) $$E=E_{\mathrm{bond}}+E_{\mathrm{angle}}+E_{\mathrm{dihedral}}+E_{\mathrm{non}-\mathrm{bonded}}$$

The parameters of the OPLS-UA force field from the Moltemplate software [21] used for the MD simulations are shown in Table 1. The carbon atoms of polyisoprene have been modeled using the parameters of the carbon atom in isobutene. The CH groups of the polymers have been simulated as CH groups of alkene. To describe the ${\mathrm{CH}}_{2}$ groups of the polymers parameters of ${\mathrm{CH}}_{2}$ group of alkanes have been used. A ${\mathrm{CH}}_{3}$ group can be modeled in 4 ways using parameters of the OPLS-UA force field. All models of the ${\mathrm{CH}}_{3}$ group have been used in MD simulations to test them. In Table 1 the parameters of the ${\mathrm{CH}}_{3}$ groups of neopentane are presented. Dihedral interactions are not explicitly considered in the OPLS-UA force field used. Coefficients for special bonds have been set to zero in all simulations.

Non-bonded interactions have been modeled only by van der Waals interactions. The van der Waals interaction has been described by a 12/6 Lennard-Jonnes potential, which is given by

(2) $$E_{\mathrm{LJ}}=\left\{\begin{array}{cc} 4\epsilon [{\left(\frac{\sigma }{r}\right)}^{12}-{\left(\frac{\sigma }{r}\right)}^{6}] & \mathrm{if}\;r\leq r_{c},\\ 0 & \mathrm{otherwise}. \end{array}\right.$$

$r_{c}$ is a cutoff distance, which has been set to 10 Å in all simulations. The parameters of the Lennard-Jonnes potential used in the MD simulations are shown in Table 1. The other parameters were calculated using the Lorentz-Berthelot mixing rules. All simulations have been carried out using the LAMMPS [11] software package.

After preparing a polymeric chain, the size of a periodic supercell has been increased to prevent interactions of the chain with its copies. A MD simulation of the chain has been performed for a NVE ensemble at *T* = 900 K with a time step of 0.2 fs applying the Langevin thermostat [22] to get a coiled structure of the polymer. The chain has been slowly cooled by applying a pressure of about 1000 atm until normal conditions in a NPT ensemble with a time step of 0.2 fs. The procedure of cooling under pressure has been done three times. Nose-Hoover’s thermostat and barostat with damping parameters 100 and 1000 time steps, respectively, have been used to reach the desired temperature, pressure and density of the simulated system.

When a chain has reached its density at normal conditions, it has been replicated three times in each direction of Cartesian coordinates. As a result, 27 chains have been considered in one periodic supercell as a polymeric system. That polymeric system was simulated in a micro-canonical ensemble using the Langevin thermostat [22] to get it’s amorphous structure, and was slowly cooled in a NPT ensemble until it reached normal conditions. Time steps and damping parameters of Nose-Hoover’s thermostat and barostat were the same as for the modeling of one chain.

Before calculating the thermal conductivity of a polymeric system at different temperatures, firstly, it was simulated for 40 ps in a NPT ensemble at the desired temperature and atmospheric pressure with a time step 0.2 fs to get the equilibrated density of the system. Damping parameters of Nose-Hoover’s thermostat and barostat were 100 and 1000 time steps, respectively. After that, the system was modeled in a NVT ensemble for 100 ps with a time step of 1 fs to equilibrate the temperature. By the Green-Kubo formula, the thermal conductivity of an isotropic material can be found as

(3) $$\lambda =\frac{V}{3k_{B}T^{2}}\int_{0}^{\infty }\;<J\left(0\right)J\left(t\right)>dt$$

where *J* is the heat flux calculated as

(4) $$J=\frac{1}{V}\left[\sum_{i}e_{i}\vec{\upsilon_{i}}-\sum_{i}S_{i}\vec{\upsilon_{i}}\right]=\frac{1}{V}\left[\sum_{i}e_{i}\vec{\upsilon_{i}}+\sum_{i<j}F_{ij}\vec{\upsilon_{j}}\vec{r_{ij}}\right]=\frac{1}{V}\left[\sum_{i}e_{i}\vec{\upsilon_{i}}+\frac{1}{2}\sum_{i<j}F_{ij}\left(\vec{\upsilon_{i}}+\vec{\upsilon_{j}}\right)\vec{r_{ij}}\right]$$

where $e_{i}$ is the total enegy of *i*-th atom, $S_{i}$ is the stress tensor of *i*-th atom, $\vec{\upsilon_{i}}$ and $\vec{\upsilon_{j}}$ are velocities of *i*-th and *j*-th atoms, $\vec{F_{ij}}$ and $\vec{r_{ij}}$ are the force and distance between *i*-th and *j*-th atoms.

Due to the discretization of time in MD simulations, the heat flux autocorrelation function turns to a sum and Equation (3) can be rewritten as follows [23]

(5) $$\lambda \left(\tau_{M}\right)=\frac{V\Delta t}{3k_{B}T^{2}}\sum_{m=1}^{M}\frac{1}{\left(N-m\right)}\sum_{n=1}^{N-m}J_{i}\left(n\right)J_{j}\left(m+n\right)$$

where $\lambda (\tau_{M})$ is the thermal conductivity obtained from summation to time step *M* (*M* = 0, 1, …, N−1), *N* is the total number of simulation steps and $\tau_{M}$ = *M*Δt.

After equilibrating density and temperature of the polymeric system, a NVE ensemble has been simulated for 1.5 ns with a time step of 1 fs. The heat flux has been calculated at each time step and the thermal conductivity has been obtained for each correlation time interval. The correlation time was 1.5 ps.

To extract the dependence of the thermal conductivity on the molecular weight of a polymeric chain as well as on the sizes of simulated box, polymer chains with a degree of polymerisation of 100, 200, 300 and 400 have been prepared. The procedure of obtaining such a polymeric system was the same as described above. Before calculating the heat flux of the polymeric system in a NVE ensemble, it has been modeled in a NPT ensemble for 40 ps and than in a NVT ensemble for 100 ps to obtain the equilibrated density and temperature of the system. The polymers used in the simulations were mixtures of cis and trans configurations.

To validate the results of the thermal conductivity derived from MD simulations, measurements of the thermal conductivity of untreated polyisoprene (LIR-50) with a molecular weight of 54,000 g/mol and untreated polybutadiene (LBR-300) with a molecular weight of 45,000 g/mol have been done. Polybutadiene (LIR-50) and polyisoprene (LBR-300) are polymers in cis and trans configurations [24]. They don’t contain vinyl configurations. These measurements have been performed using the transient hot wire method.

The transient hot wire approach is based on solving Fourier’s heat equation for isotropic materials. In a mathematical model it is assumed, that a linear wire embedded in the material is infinitely thin and infinitely long. A perfect contact between the wire and the sample is required. The thermal conductivity of the material can be found from the temperature change and the power of heat generation of the wire and is calculated as follows [25]:

(6) $$\Delta T=\frac{q}{4\pi \lambda }\ln\frac{4kt}{a^{2}C}$$

where *q* is the heat generation power per unit length of the wire, λ is the thermal conductivity of the sample, *a* is the radius of the wire, *k* is the thermal diffusivity of the sample, *t* is the time after the beginning of the heating of the wire, *C* = expγ, where γ is Euler’s constant. If one takes a difference between the change of temperature between two different points in time, Equation (6) can be written as

(7) $$\lambda =\frac{UI\ln\frac{t_{2}}{t_{1}}}{4\pi l(T_{2}-T_{1})}$$

where *U* is the voltage between the ends of the wire, *I* is the current through the wire and *l* is its length.

In all experiments, an Alumel wire has been used as heating element and as thermocouple to measure the temperature change of the polymer samples. The temperature change has been measured via the change of the wire’s resistance, which linearly depends on the temperature and has been measured using a Wheatstone bridge.

## 3. Results and Discussion

**Figure 2:**
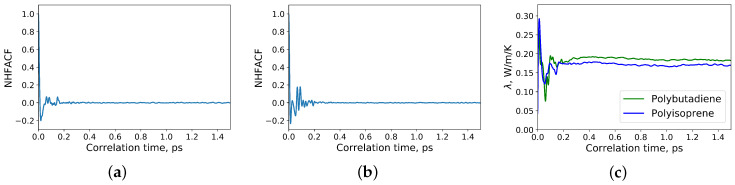
(**a**) Normalized heat flux autocorrelation function (NHFACF) of polyisoprene; (**b**) normalized heat flux autocorrelation function (NHFACF) of polybutadiene; (**c**) thermal conductivities of polyisoprene and polybutadiene at 22 °C and atmospheric pressure.

The normalized heat flux autocorrelation functions (NHFACF) and dependence of the thermal conductivity of the polymers from correlation time are presented in Figure 2. As shown in Figure 2, a correlation length of 1.5 ps was sufficient to obtain a drop to zero for the heat flux autocorrelation functions. The data for the analysis were taken from the last correlation time interval. The heat flux autocorrelation functions and thermal conductivities along each direction were compared. It was observed, that they are equal in all directions. Thus, the polymers of the simulations were isotropic like those of the measured samples.

**Figure 3:**
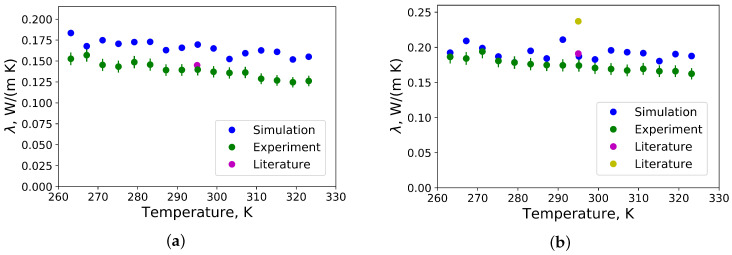
(**a**) Thermal conductivities of polyisoprene as function of temperature. For comparison, literature data from [18] are taken; (**b**) Thermal conductivity of polybutadiene as function of temperature. For comparison, literature data from [17] are taken: magenta and yellow circles indicate thermal conductivities of medium vinyl content polybutadiene with a molecular weight of 2600 g/mol and highly vinyl content polybutadiene with a molecular weight of 100,000 g/mol, respectively.

The thermal conductivity of polyisoprene and polybutadiene in a temperature range from −10 °C to 50 °C received from MD simulations by the Green-Kubo approach as well as from measurements by the transient hot wire method are presented in Figure 3. In comparison with available literature data at 22 °C, a value of λ = 0.14 W/m/K has been found for the thermal conductivity of polyisoprene from measurements with the transient hot wire method, which is close 0.134 W/m/K [26] and 0.145 W/m/K [18]. Our calculated value of the thermal conductivity from MD simulations is λ = 0.17 W/m/K, which is more accurate than the results from other available literature data from MD simulations λ = 0.35 W/m/K [4] and λ ≈ 0.06 W/m/K [6]. The dependence of the thermal conductivity on the temperature is in agreement with experimental data. In comparison with ref. [19], where it has been found, that thermal conductivities of mixtures of crosslinked cis and trans polyisoprene are higher than thermal conductivities of pure crosslinked cis and trans polyisoprenes, our samples were not crosslinked.

The slight overestimation of the thermal conductivity of polyisoprene can be explained by the force field parameters used for ${\mathrm{CH}}_{3}$ group, which are probably not accurate enough for polyisoprene. Available parameters to describe interactions between the atoms from the OPLS-UA force field for a ${\mathrm{CH}}_{3}$ group have been tested, such as from *n*-alkane, ethane, isobutane and neopentane. The most accurate results for the density and the thermal conductivity of polyisoprene have been achieved, when parameters of neopentane like ${\mathrm{CH}}_{3}$ group have been taken to represent ${\mathrm{CH}}_{3}$ groups of polyisoprene, which agrees with the chemical environment. Another reason can be the consideration of degrees of freedom, which are not excited in reality. For instance, the thermal conductivity of polyamide-6,6 linearly depends on the number of degrees of freedom per polymeric chain [7]. In this research four types of force fields have been considered, and the best accuracy of the thermal conductivity has been obtained by the fully constrained united atom force field. Using partially or fully constrained united atom force fields, this can increase the accuracy of prediction of thermal conductivities from MD simulations.

The thermal conductivity of polybutadiene has been found from measurements lower than for a medium vinyl content polybutadiene with a molecular weight of 2600 g/mol and highly vinyl content polybutadiene with a molecular weight of 100,000 g/mol [17]. Such a deviation can be explained by differences in the chemical structure and in the molecular weight.

**Figure 4:**
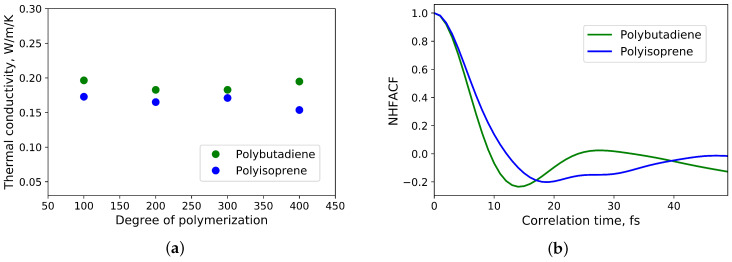
(**a**) Dependence of thermal conductivity of polyisoprene and polybutadiene from the degree of polymerisation and number of simulated atoms; (**b**) the first minima of the normalized heat flux autocorrelation functions of polyisoprene and polybutadiene.

In Figure 4a the influence of the degree of polymerization and thus also the number of simulated atoms on the thermal conductivity is shown. The results are in agreement with [3]. Based on this work, the heat transport in untreated polyisoprene and polybutadiene takes place mainly via phonons. In addition, 27 chains consisted of 200 monomer units are enough to eliminate size effects for the calculation of the thermal conductivity.

To analyse the normalized heat flux autocorrelation functions, one can use the one-dimensional harmonic oscillator model as it was done in [6]. The first minimum (see Figure 4b) of the normalized heat flux autocorrelation function of polyisoprene is located at *t*≈ 18 fs, which corresponds to a wavenumber of $\bar{\nu }$ ≈ 295 ${\mathrm{cm}}^{-1}$. It is lower than C-C-C deformation vibrations ($\bar{\nu }$ ≈ 390 ${\mathrm{cm}}^{-1}$) and higher than C-C torsion vibrations of cis-1,4-polyisoprene ($\bar{\nu }$ ≈ 223 ${\mathrm{cm}}^{-1}$) [27]. For polybutadiene the first minimum (see Figure 4b) of the normalized heat flux autocorrelation function is located at *t* ≈ 14 fs. It matches to a wavenumber of $\bar{\nu }$ ≈ 379 ${\mathrm{cm}}^{-1}$ and is located in the vicinity of wavenumbers, that correspond to C-C-C deformations vibrations of cis-1,4-polybutadiene ($\bar{\nu }$ ≈ 405 ${\mathrm{cm}}^{-1}$) and trans-1,4-polybutadiene ($\bar{\nu }$ ≈ 439 ${\mathrm{cm}}^{-1}$) [28]. As a result, the heat in this polymers is mainly transferred by low frequency phonons. This is in agreement with [3]. Consequently, the main mechanism of heat transfer in untreated polyisoprene and polybutadiene is based on phonon transport.

## 4. Conclusions

For the first time, the thermal conductivities of untreated polyisoprene and polybutadiene were calculated by MD simulations and measured by the transient hot wire method in the temperature range from −10 °C to 50 °C at atmospheric pressure. In case of polyisoprene, MD simulations using the Green-Kubo approach can predict the influence of the temperature on thermal conductivity with a slight offset. Four types of force field parameters for ${\mathrm{CH}}_{3}$ groups of polyisoprene were tested. The best accuracy concerning density and thermal conductivity have been achieved with parameters of neopentane from the OPLS-UA force field. An overestimation of the thermal conductivity by the OPLS-UA force field is in average 0.026 W/m/K. These results are more accurate in comparison with previous MD simulations of polyisoprene in literature. For polybutadiene, the results of MD simulations are in good agreement with experimental data. From analysis of normalized heat flux autocorrelation functions of the polymers, it has been found, that the main mechanism of heat transfer in these polymers is by transport of low frequency phonons, which agrees well with literature data for other polymers. Finally, it can be concluded, that this approach is thus adequate to describe new polymer blends in the future. MD simulations using the Green-Kubo approach and OPLS-UA force field can be used for calculations of the thermal conductivities of rubbers, which are based on polyisoprene and polybutadiene.

## Figures and Tables

**Table 1:** Force field parameters used in MD simulations. Usual factor of 1/2 in stretching and bending interactions is included in *K*.

Force Field Parameters for Polyisoprene			Force Field Parameters for Polybutadiene		
$E_{\mathrm{bond}}=K{\left(r-r_{0}\right)}^{2}$	$K[\frac{\mathrm{kcal}}{\mathrm{mol}\;{Å}^{2}}]$	$r_{0}\left[Å\right]$	$E_{\mathrm{bond}}=K{\left(r-r_{0}\right)}^{2}$	$K[\frac{\mathrm{kcal}}{\mathrm{mol}\;{Å}^{2}}]$	$r_{0}\left[Å\right]$
C−${\mathrm{CH}}_{2}$	317	1.5			
C−${\mathrm{CH}}_{3}$	317	1.5	CH=CH	530	1.34
C=CH	530	1.34	CH−${\mathrm{CH}}_{2}$	317	1.5
CH−${\mathrm{CH}}_{2}$	317	1.5	${\mathrm{CH}}_{2}$−${\mathrm{CH}}_{2}$	260	1.526
${\mathrm{CH}}_{2}$−${\mathrm{CH}}_{2}$	260	1.526			
$E_{\mathrm{angle}}=K{\left(\theta -\theta_{0}\right)}^{2}$	$K[\frac{\mathrm{kcal}}{\mathrm{mol}\;{\mathrm{rad}}^{2}}]$	$\theta_{0}\left[\mathrm{degrees}\right]$	$E_{\mathrm{angle}}=K{\left(\theta -\theta_{0}\right)}^{2}$	$K[\frac{\mathrm{kcal}}{\mathrm{mol}\;{\mathrm{rad}}^{2}}]$	$\theta_{0}\left[\mathrm{degrees}\right]$
${\mathrm{CH}}_{2}$−C−${\mathrm{CH}}_{3}$	70	124			
${\mathrm{CH}}_{2}$−C=CH	70	118	${\mathrm{CH}}_{2}$−CH=CH	70	118
${\mathrm{CH}}_{3}$−C=CH	70	118			
$E_{\mathrm{LJ}}=4\epsilon \left[{\left(\frac{\sigma }{r}\right)}^{12}-{\left(\frac{\sigma }{r}\right)}^{6}\right]$	$\epsilon [\frac{\mathrm{kcal}}{\mathrm{mol}}]$	σ[Å]	$E_{\mathrm{LJ}}=4\epsilon \left[{\left(\frac{\sigma }{r}\right)}^{12}-{\left(\frac{\sigma }{r}\right)}^{6}\right]$	$\epsilon [\frac{\mathrm{kcal}}{\mathrm{mol}}]$	σ[Å]
${\mathrm{CH}}_{3}$−${\mathrm{CH}}_{3}$	0.145	3.96			
${\mathrm{CH}}_{2}$−${\mathrm{CH}}_{2}$	0.118	3.905	${\mathrm{CH}}_{2}$−${\mathrm{CH}}_{2}$	0.118	3.905
CH−CH	0.115	3.8	CH−CH	0.115	3.8
C−C	0.105	3.75			
